# Vacuum Compression Molding as a Screening Tool to Investigate Carrier Suitability for Hot-Melt Extrusion Formulations

**DOI:** 10.3390/pharmaceutics12111019

**Published:** 2020-10-24

**Authors:** Gauri Shadambikar, Thomas Kipping, Nicole Di-Gallo, Alessandro-Giuseppe Elia, Anja-Nadine Knüttel, Daniel Treffer, Michael. A Repka

**Affiliations:** 1 Department of Pharmaceutics and Drug Delivery, University of Mississippi, Oxford, MS 38677, USA; marepka@olemiss.edu; 2 Merck KGaA, Frankfurter Str. 250, 64293 Darmstadt, Germany; thomas.kipping@merckgroup.com (T.K.); nicole.di-gallo@merckgroup.com (N.D.-G.); alessandro-giuseppe.elia@merckgroup.com (A.-G.E.); anja-nadine.knuettel@merckgroup.com (A.-N.K.); 3 MeltPrep GmbH, Nikolaiplatz 4/3, 8020 Graz, Austria; daniel.treffer@meltprep.com

**Keywords:** polyvinyl alcohol, screening tool, hot-melt extrusion, amorphous solid dispersion, formulation development

## Abstract

Hot-melt extrusion (HME) is the most preferred and effective method for manufacturing amorphous solid dispersions at production scale, but it consumes large amounts of samples when used for formulation development. Herein, we show a novel approach to screen the polymers by overcoming the disadvantage of conventional HME screening by using a minimum quantity of active pharmaceutical ingredient (API). Vacuum Compression Molding (VCM) is a fusion-based method to form solid specimens starting from powders. This study aimed to investigate the processability of VCM for the creation of amorphous formulations and to compare its results with HME-processed formulations. Mixtures of indomethacin (IND) with drug carriers (Parteck^®^ MXP, Soluplus^®,^ Kollidon^®^ VA 64, Eudragit^®^ EPO) were processed using VCM and extrusion technology. Thermal characterization was performed using differential scanning calorimetry, and the solid-state was analyzed via X-ray powder diffraction. Dissolution studies in the simulated gastric fluid were performed to evaluate the drug release. Both technologies showed similar results proving the effectiveness of VCM as a screening tool for HME-based formulations.

## 1. Introduction

In 1971, hot-melt extrusion (HME) was introduced as a formulation technology platform for the pharmaceutical industry [1]. Neither solvents nor complicated processing steps are required in HME to formulate a specialized drug delivery formulation. It can be used for the formulation of various drug delivery systems, but one of its significant uses is to improve the solubility of poorly soluble drugs [2]. This is one of the key challenges in today’s research in formulation and development. Most of the new drugs under development have poor solubility of the active pharmaceutical ingredients [3]. The solubility and permeability of a drug are categorized by the biopharmaceutical classification system (BCS) into four classes based on its aqueous solubility and intestinal permeability [4]. The percentage of new molecular entities with poor solubility is likely to increase due to development in combinatorial chemistry and the significant importance of lipophilic receptors [5]. The creation of amorphous solid dispersions (ASD) is one way of formulating such poorly soluble drugs [6]. The solubility and bioavailability can be improved by orders of magnitudes via HME [7]. The solid-state of the poorly soluble crystals is changed via HME. They are dissolved within the polymer matrix when processed in the molten state. Once cooled down, the individual molecules are entrapped within the polymer matrix, forming a dispersed solid solution. When the solid solution is dissolved, the polymer matrix controls the dissolution rate and releases the individual molecules of the active substance. High supersaturation levels can be maintained over a long time when suitable excipients are selected. Novel generations of amorphous solid dispersions are aiming to improve the dissolution profile of the carrier matrix by applying ingredients that provide additional surface activity or self-emulsifying properties [8]. Despite this advantage, it can also increase the risk of recrystallization during storage. Amphiphilic polymers can provide a relevant advantage, as no additional excipients are required to ensure the supersaturation of low soluble compounds. 

Even with increasing demand, HME lacks reliable tools for formulation development, which allows access to reliable material data on a small scale. The HME technology was developed for processing plastics, and most of the development efforts were put on efficiency and maximizing the throughput [9]. When it was brought into the pharmaceutical labs, the smallest plastic extruders were adapted to suit the pharmaceutical manufacturing practices. To address the need to perform small-scale screening, the equipment was downsized in dimensions or its concept slightly changed to reduce the minimum amount of material required to generate initial results [10]. Small-scale extruders require at least a few grams of a material to allow first fusion-based investigations. The large fraction of the material, however, is lost during the startup phase or remains in the dead zone of the extruder. Formulation development with new chemical entities (NCEs) with this limitation is impractical as the available quantity of the Active Pharmaceutical Ingredient (API) is the limiting factor. Thus, to develop HME-based formulations comprising NCE, material sparing selection tools are necessary [11]. The HME technology is already quite established with several products like Lacrisert^®^, Kaletra^®^, Nucynta^®^, NuvaRing^®^, and Zythromax^®^ in the market [12]. 

### 1.1. Hot-Melt Extrusion

Very few articles are published with the use of HME in first stage investigations. Some reports use a combined approach of hot stage microscopy and 5 mm twin-screw extruders in their study to observe the change in crystal form and dissolution of the sample under the influence of the temperature for the development of scale-up [11,13]. The development of implants by hot-melt extrusion necessitated the use of small-scale extruders with small batch sizes. For the predictive formulation of protein-loaded implants, a 9-mm twin-screw extruder was used [14]. To determine operational and performance qualification on mixing in solid dispersion preparation in early-stage HME development, a conical twin-screw extruder was utilized [15]. 

### 1.2. Differential Scanning Calorimetry

Differential scanning calorimetry (DSC), an established analytical method, is one of the many thermal methods used as a screening tool to predict the drug-polymer solubility [16]. Experimental DSC data was used to predict a drug-polymer phase diagram and miscibility for Felodipine and polyacrylic acid [17]. Knopp et al. predicted drug-polymer solubility at elevated temperatures from DSC data for binary systems of five model drugs with PVP and PVA [18]. Similarly, for HME formulations, the solubility of crystalline drugs in the polymer was determined by a combined approach of DSC measurements and a reliable mathematical algorithm to determine a complete solubility of a drug in a polymer [19]. The solubility measurements for the compounds with very low absolute solubilities or exhibiting small changes in solubilities with temperature cannot give reliable results when analyzed by DSC [20]. 

### 1.3. Solvent Casting

Solvent casting is usually applied to generate the first insights into a new API/carrier formulation. However, for a fusion-based product via HME, its results might deviate. A recent publication combines solvent casting with a simplified extrusion step to become a fusion-based screening method [21]. Novel approaches are also combining rapid solvent evaporation methods with an additional heating step, which is quite successful in estimating formulation performance [22]. To utilize a minimum amount of drug, some research reports a high throughput screening technology developed by utilizing a 96 well plate system to identify optimal drug load and polymer using a solvent casting method [23]. 

One drawback of solvent casting is to find a common solvent where the polymers and drug substances together will be solubilized. Especially for hydrophilic polymers, this proves to be a challenge.

### 1.4. Vacuum Compression Molding

**Figure 1:**
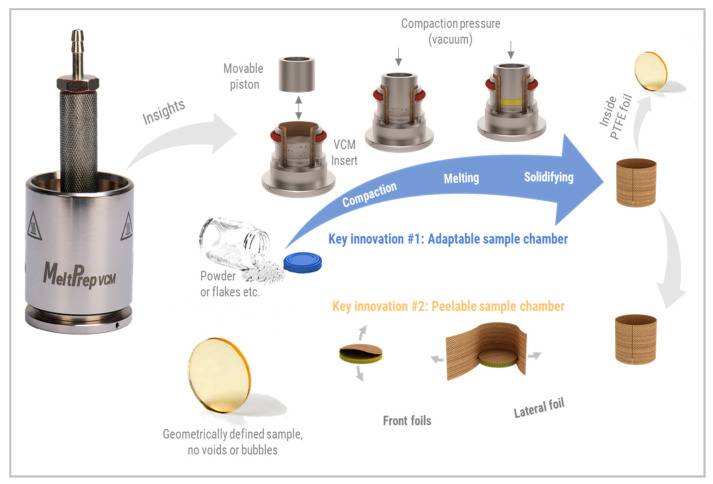
MeltPrep^®^ Vacuum Compression Molding (VCM) process.

To overcome the above limitation of established screening tools, a novel approach to screen the new chemical entity with the polymer can be done using Vacuum Compression Molding (VCM). VCM is a fusion-based method to form solid specimens starting from powders (Figure 1). The process was first introduced in 2014 for sample preparation for rheological measurements of pharmaceutical polymers [24]. For the rheological measurement discs with 25 mm were introduced.

The centerpiece of the VCM Process is the sample chamber. It is a chamber that is fully lined by PTFE foils and still adaptable in volume. The arrangement of three separation foils forms the PTFE lining. One lateral foil covers the inner cylindrical surface of the VCM chamber, and two circular front foils cover the top and the bottom surface of the chamber. The starting material in powder form is filled inside the chamber formed by the foils. Once a vacuum is applied, the piston is pressed with 150N on the sample, which compacts the powder bed. The VCM Tool is subsequently placed on the hot plate of the VCM Essential to warm and then fuse the particles to a homogeneous sample. Since everything occurs under a vacuum within the tool, no air bubbles are entrapped within the polymer melt. The VCM Tool is quenched on the cooling unit by contact cooling and convection by a directed air cooling. The sample can be easily released from the chamber as the PTFE foils have excellent non-stick behavior and can be peeled off like a sticker from the sample. The method works without macroscopic mixing as only minimal mechanical mixing occurs during the processing when particles fuse. Larger particles in the millimeter and sub-millimeter range will not form homogeneous results. They require diffusion as the dominant mixing mechanism to obtain extrusion-like results. Hence, preconditioning of the powder before VCM processing is required when formulations with several components are molded to obtain homogeneous samples. The preconditioning can be obtained via cryogenic milling as it can alter the polymorphic structures [25]. 

The processing towards a solid form devoid of air inclusions was not possible in a lossless manner before the introduction of VCM. The VCM process has attracted the attention of several research groups and has been successfully applied to the screening of HME formulations like multilayer intravaginal rings [26]. Another group was speeding up the development of abuse-resistant formulations [27]. Evans et al. used VCM for material characterization to determine the solid density of API-loaded formulations, which was subsequently fed into a 1D simulation software of extruders to predict processing behavior best possible [28]. Since VCM results in samples with a defined surface area which corresponds to the cylinder surface, it enables intrinsic dissolution testing. It allows eliminating surface effects on the dissolution tests. The dissolution of polymer formulations is a complex topic and is described in several articles [29,30,31]. Insights can be obtained by dissolution measurements on samples with defined geometries. Dissolution mechanisms like surface erosion, bulk erosion, swelling, or diffusion behavior can be studied on quickly accessible VCM samples. Dissolution tests on the VCM samples enable direct performance comparison. Results can be used to tailor the particle size of a final dosage form. If powder or granules are required for the development when a conventional compacted tablet is desired, VCM samples of example 2–5 g per batch can be milled afterwards and used for the development tasks.

The objective of the study was to investigate the VCM processability for ASD and to compare its results with HME-processed formulations. Mixtures of indomethacin (IND) with drug carriers (Parteck^®^ MXP, Soluplus^®^, Kollidon^®^ VA 64, Eudragit^®^ EPO) were processed using VCM and extrusion technology. From the literature, it was found that indomethacin can form a stable ASD with Soluplus^®^, Kollidon^®^ VA 64, and Eudragit^®^ EPO. These excipients were able to enhance the solubility and inhibit crystallization for indomethacin. Until now, the interaction of Parteck^®^ MXP and indomethacin has not yet been investigated. This paper gives insight into ASD using Parteck^®^ MXP. The thermal characterization of the ASD was performed using differential scanning calorimetry and X-ray powder diffraction. The drug release performances were evaluated in simulated gastric fluid [32,33,34]. 

## 2. Materials and Methods 

### 2.1. Materials

Indomethacin was obtained from Sigma-Aldrich (St. Louis, MO, USA). Parteck^®^ MXP was purchased from EMD Millipore Sigma (Darmstadt, Germany). Soluplus^®^ and Kollidon^®^ VA-64 were purchased from BASF (Ludwigshafen, Germany). Eudragit^®^ EPO was purchased from Evonik Industries (Essen, Germany). The marketed products (Indo-CT 50 mg capsules) were purchased from AbZ Pharma (Ulm, Germany). All other reagents were of either high-performance liquid chromatography (HPLC) or analytical grade.

#### 2.1.1. Active Pharmaceutical Ingredient (API)

The model API used in this study was indomethacin (IND) which is a BCS class II compound. Its physicochemical properties are summarized in Table 1. Indomethacin is a potent non-steroidal anti-inflammatory drug [35]. It is poorly water-soluble with low glass transition temperature and thermally stable, making it a good choice as a model drug for amorphous solid dispersions [36]. 

#### 2.1.2. Carriers

The excipients selected for the present study were Kollidon^®^ VA 64 a copovidone-(vinylpyrrolidone-vinyl-acetate), Soluplus^®^ a graft copolymer (polyvinyl-caprolactam-polyvinyl-acetate-polyethylene-glycol), Parteck^®^ MXP (polyvinyl-alcohol), and Eudragit^®^ EPO (poly-methyl-methacrylate). The physicochemical properties of the excipients are mentioned in the Table 2.

#### 2.1.3. Marketed Drug Product for Reference

Indo-CT 50 mg capsules were used as a reference in the release study. They are 50 mg hard capsules with indomethacin as the active ingredient. It is used for symptomatic treatment of pain and inflammation.

### 2.2. Processing Methods

#### 2.2.1. Preparation of Cryo-Milled Mixtures for VCM Preparation-Preconditioning of the Samples

Polymer–Indomethacin (30% *w/w*) binary mixtures were weighed and then mixed in a Turbula^®^ Mixer (Willy A. Bachofen AG, Muttenz, Switzerland) for 5 min. The mixture was then cryo-milled with liquid nitrogen in Ultra-Centrifugal Mill ZM-200 (Retsch GmbH, Haan, Germany) at 18,000 rpm and sieved through a mesh size of 250 µm.

#### 2.2.2. Vacuum Compression Molding (VCM)

The MeltPrep^®^ VCM Tool consisted of a sample holder, piston, and lid. The sticking of the sample during the preparation was prevented using separation foils. The sample holder was connected to a vacuum source. The cryo-milled mixture was filled into the sample holder which provided amorphous samples of 8 mm and 20 mm in diameter. The 8mm discs were intended to provide intrinsic-like dissolution, and the 20 mm discs were further processed via milling to create enough material to allow a direct comparison between milled extrudate and milled VCM material. The piston provided the pressure on the sample, which was heated on the hot plate until a homogenous mixture was obtained. The heating process was followed by rapid cooling to get the final product. Table 3 summarizes the parameters from MeltPrep^®^ samples with 20 mm and 8 mm in diameter. Target temperatures and respective heating times were adapted and optimized for the individual polymers. 

Samples produced via VCM have a defined geometry and yield transparent glasslike discs once amorphous systems are obtained. The circular cross-section for 25 mm discs, as it was initially introduced, is 490 mm^2^ so that for materials with densities of around 1 g/cm^3^, 1 g is enough to obtain a solid disc with a height of around 2 mm. The VCM process has attracted the attention of several pharmaceutical research groups quickly as it offers extrusion-like results and the required material amount is comparatively lower to the existing extrusion methods.

The amounts can be drastically reduced further by simply reducing the disc dimensions to smaller diameters, as it will be shown during the study. This small disc’s diameter (e.g., 2 or 5 mm) enables sample preparation of small quantities starting at ranges of 10 mg and, in addition,, an opportunity to meet similar material demands compared to solvent casting methods. 

#### 2.2.3. Hot-Melt Extrusion (HME)

**Figure 2:**
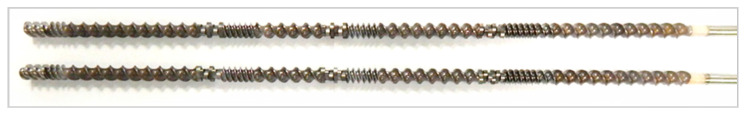
Screw configuration for Thermo Fisher Scientific^®^ Pharma 11-mm extrusion.

For the extrusion process, the pre-blended physical drug-polymer mixture was fed into the hopper. Each of these resultant drug-polymer binary mixtures containing 30% *w/w* of the drug was extruded utilizing an 11 mm co-rotating Pharma twin-screw extruder and Congrav twin-screw feeder (Thermo Fisher Scientific^®^, Waltham, MA, USA) equipped with a 2-mm round opening die. Differential scanning calorimetry was utilized to determine the extrusion processing temperature range. The target temperature for extrusion was above the melting point of indomethacin. For Soluplus^®^, Kollidon^®^ VA 64 and Eudragit^®^ EPO were at about 160 °C, anticipating the additional heat impact due to shear forces. For Parteck^®^ MXP, the temperature profile needed to be increased to overcome the polymer’s semi-crystalline alignment. Hence, the extrusion temperature was set at 190 °C. A standard screw configuration consisting of conveying and kneading elements was used (Figure 2) at a screw speed of 200 RPM. A constant feed rate of 0.2 kg/h was employed for all formulations. The process parameters were recorded, and the conditions of the steady-state operation are given in Table 4.

### 2.3. Characterization Methods

#### 2.3.1. Physical Characterization of the VCM Samples

The VCM samples were characterized for the uniformity of weight using a Mettler Toledo^®^ analytical balance. The before and after sample weights were statistically assessed via a two-tailed paired *t*-test (IBM^®^ SPSS^®^ Statistics 25, Chicago, IL, USA, 2017). The significance threshold was set at a *p*-value of 0.05. The samples were visually inspected for transparency, potential recrystallization, and bubbles.

#### 2.3.2. Differential Scanning Calorimetry (DSC)

The thermal properties of the polymer excipients, API, and samples prepared in the present study were investigated using a DSC 3+ differential scanning calorimeter (Mettler-Toledo^®^, Giessen, Germany). Nitrogen was used as the purge gas at a flow rate of 50 mL/min. For sample analysis, 5–7 mg samples were accurately weighed and sealed inside aluminum pans. The lids were pierced by the autosampler before the measurement was initiated. For thermal characterization, pure indomethacin and the milled VCM and HME materials were heated at 10 °C/min from 25 to 300 °C. Results were analyzed using the STARe SW 16.00 software (Mettler-Toledo^®^, Giessen, Germany).

#### 2.3.3. PXRD Analysis

PXRD patterns were recorded on a Stoe StadiP^®^ 611 instruments (Stoe, Germany) equipped with a Cu radiation source (*λ* = 1.54 Å) and a Mythen1K Si-strip detector. Measurements were conducted in transmission and at an acceleration voltage of 40 kV and a current of 40 mA. Scanning was performed over an angle range of 2*θ* at a step size of 0.015° and dwell time of 0.5 s. The results were analyzed using the Powdat software.

#### 2.3.4. Dissolution

The dissolution tests were carried out for the marketed product and indomethacin-loaded samples prepared using VCM and HME. Prior to the test, the 20 mm VCM and HME samples were milled. An equivalent of 50 mg of indomethacin-milled samples were used for dissolution. The 8 mm VCM discs which were equivalent to 50 mg of indomethacin were used as an entire disc. The marketed formulation (Indo-CT 50 mg) was used as a reference. The dissolution tests were carried out using the Sotax AT7 smart (Sotax, Germany) dissolution tester following the USP apparatus 2 method. The dissolution medium was 900 mL of simulated gastric fluid (SGF) at 37.5 ± 0.5 °C with a stirring rate of 75 rpm. SGF was selected as a dissolution media to determine solubility of indomethacin in stomach on oral administration. Three replicates were performed for each sample. The amount of indomethacin dissolved was determined using the online UV–VIS method at 318 nm.

## 3. Results

### 3.1. Vacuum Compression Molding

**Figure 3:**
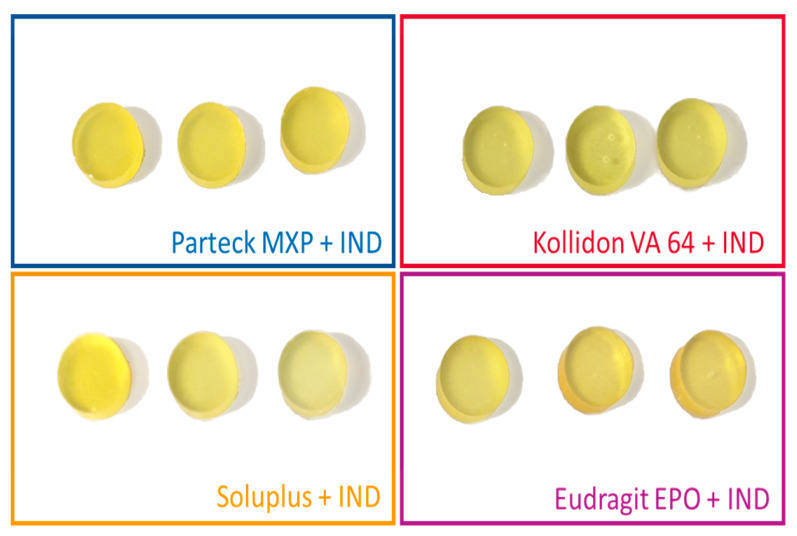
Images of 8 mm VCM disc samples loaded with 30% indomethacin (IND) (20 mm diameter not shown, similar in appearance).

Images of the produced VCM discs are shown in Figure 3. The VCM discs had a defined cylindrical geometry and a yellowish appearance with all polymers. All discs were transparent, no crystals were visible, indicating that indomethacin was dissolved in the carrier materials. The Kollidon^®^ VA 64 samples occasionally showed some bubbles, which might be related to residual moisture of the material. Compared to extrusion temperature readings, VCM requires slightly higher hot plate temperatures to compensate for non-existing shear heating. It does not mean that VCM requires higher processing temperatures compared to the macroscopic extruder temperature readings, as the readouts of the extruder do not capture local shear heating within the polymer melt. Temperature increases of 20 °C or even more are typical values obtained by simulations conducted to evaluate shear heating during twin-screw extrusion [41]. Table 5 shows the lossless preparation of the VCM samples produced for a filling weight of 167 and 500 mg for 8 mm and 20 mm, respectively. The resulting VCM samples were showing that no material was lost during VCM processing. This was statistically analyzed by comparing before and after weights for each corresponding polymer mixtures with a two-tailed paired *t*-test with a confidence interval of 95%. A *p*-value of more than 0.05 was obtained for each of the comparisons indicating that the difference is non-significant. The visual inspection indicated amorphization, and the samples were further analyzed in the subsequent analysis sections. The VCM samples (*n* = 3 for each dimension), as used to screen one formulation, required less than 2 g of starting material, which corresponds to 1/5 of the 3-min flushing time of the small-scale extruder chosen for this study. Further, potential downscaling using small-scale analysis tools will also allow using smaller VCM tools, e.g., with 2 mm diameter, making screenings in the mg-scale feasible.

### 3.2. Hot-Melt Extrusion

The extrusion was started under the parameters mentioned in Section 2.2.3. All evaluated polymers were processed at the identified parameters. The obtained extrudates were pelletized using a Brabender strand pelletizer (Brabender GmbH and Co. KG, Duisburg, Germany) with a pellet size of 2mm and further milled for all the evaluations (IKA tube mill 100, Staufen, Germany) to provide a similar particle size compared to the material used for a fast disintegration tablet. All the polymers utilized showed good extrudability under the utilized processing temperatures.

### 3.3. Differential Scanning Calorimetry

**Figure 4:**
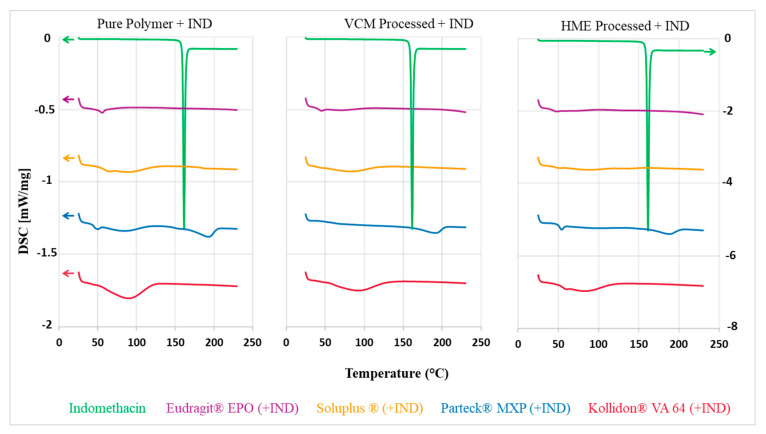
DSC thermogram left: neat polymers and IND; middle: milled VCM material from processed mixtures of polymers and IND (20 mm); right: milled HME material from processed mixtures of polymers and IND; heat flow offset used for better visibility.

Thermal analytical techniques provide data about thermal stability, melting point, and recrystallization temperatures [32]. The sharp endothermic peak at 162 °C corresponds to the melting point of indomethacin (Figure 4). Parteck^®^ MXP, Soluplus^®,^ Kollidon^®^ VA 64, and Eudragit^®^ EPO showed T_g_ (glass transition) temperatures of 50 °C, 70 °C, 101 °C, and 57 °C, respectively, confirming their amorphous state (Figure 4 left). An endothermic melting peak at 180 °C was observed for the Parteck^®^ MXP indicating its semi-crystalline nature caused by an alignment of the linear polymer chains. 

Indomethacin was completely miscible at the concentration of 30% *w/w* in the polymer-carriers, which is an important prerequisite to attain a solid dispersion. A characteristic melting endothermic indomethacin peak was absent in the VCM and HME formulations (Figure 4 middle and right). Thus, it confirmed that the indomethacin was present in an amorphous state in the VCM discs and melt-extruded formulations. The semi-crystalline peak for Parteck^®^ MXP is still observed in the VCM formulations, proving its semi-crystalline nature which is unaffected by the VCM and HME processing. 

### 3.4. PXRD Analysis

DSC studies indicated drug-polymer miscibility in the HME and VCM formulations. However, DSC measurements have limited sensitivity of measuring crystalline residuals within the material. Therefore, the solid-state of the formulations was further investigated by the PXRD analysis.

**Figure 5:**
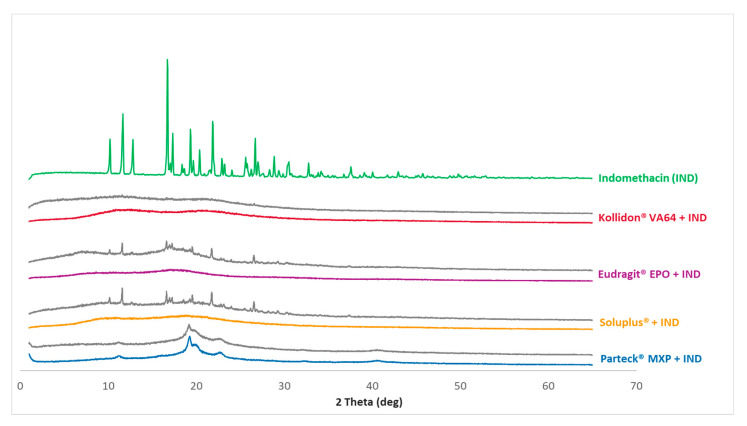
PXRD measurements: The curves for the formulations are clustered. In front of each cluster are the VCM-processed samples with a color code used throughout the data; and in the back are the cryo-milled physical mixtures for each polymer and indomethacin depicted in gray. A pure indomethacin diffractogram for reference purpose is given in the back as a single curve.

**Figure 6:**
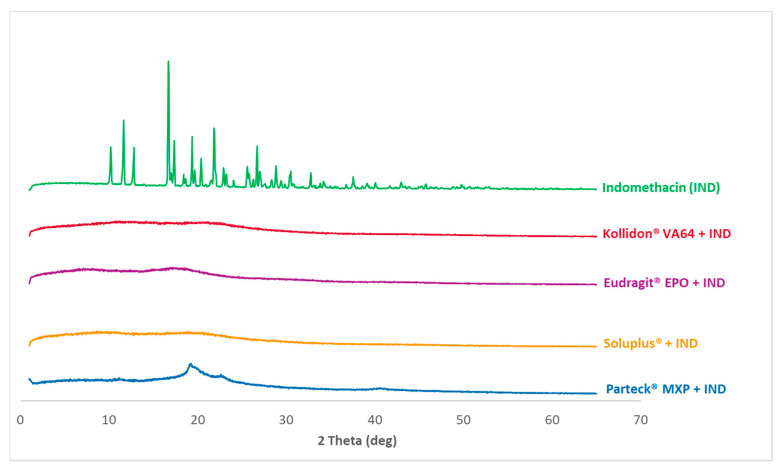
PXRD measurements: Diffractograms for processed HME samples and pure indomethacin diffractogram for reference purpose is given in the back as a single curve.

Figure 5 shows the diffractograms of the measured data of processed VCM samples as well as the cryo-milled physical mixtures of each polymer and indomethacin. Figure 6 shows the diffractograms of processed HME samples. The reference data of pure indomethacin show a significant crystalline peak confirming the crystalline state of the starting material.

XRD pattern for the milled extrudate and milled VCM samples showed a complete absence of characteristic crystalline peaks of indomethacin. Both melt processing routes (HME and VCM) deliver comparable results. In contrast, Parteck^®^ MXP shows a broad halo between 2θ of 19°–25° confirming the semi-crystalline nature of PVA polymer.

The solid-state of the cryo-milled mixtures, as seen in Figure 5, is already influenced via the cryo-milling process. The effect of cryo-milling on polymorphic transformation has been reported earlier for indomethacin [42]. The formation of an amorphous state upon cryo-milling is because of the continuous disordering process of the indomethacin lattice [43]. This observed amorphization in indomethacin is a cryo-milling time-dependent process, and it has been extensively studied and reported [43,44]. In the case of Parteck^®^ MXP and Kollidon^®^ VA 64, this effect is seen to be more pronounced than in Eudragit^®^ EPO and Soluplus^®^. As cryo-milling was not the objective of the study, the observed differences in the amorphization of the physical mixtures were not further explored.

When VCM screening is applied, cryo-milling is beneficial since it provides uniform mixing and size reduction for the physical mixture. The short cryo-milling as preconditioning decreases the physical mixtures crystallinity by bringing the path length (particle dimensions) down to small length scales that can make diffusion as the main mixing mechanism. The VCM process can achieve the full amorphization of the subjected formulation without stressing the material.

### 3.5. Dissolution

**Figure 7:**
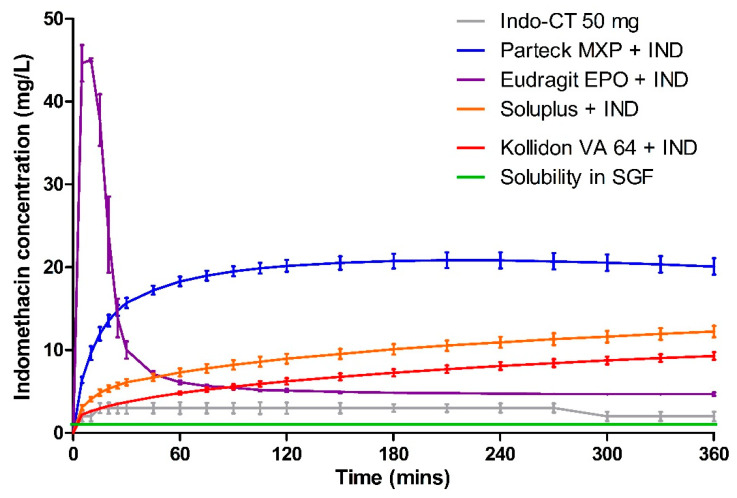
Dissolution testing for milled VCM material in simulated gastric fluid.

**Figure 8:**
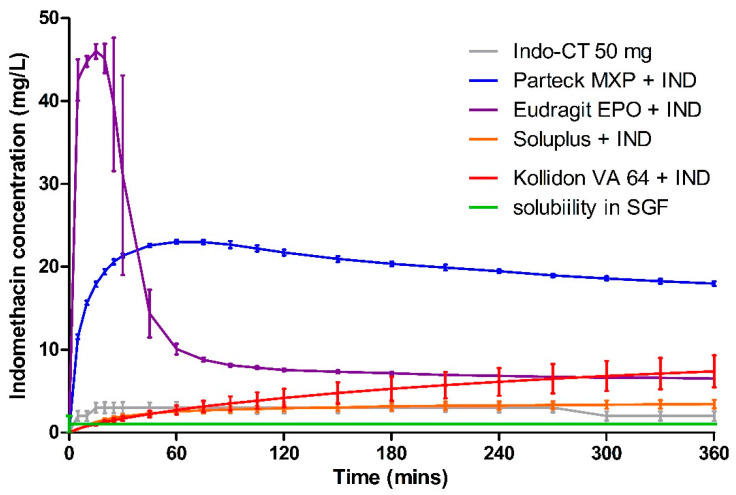
Dissolution testing for milled HME material in simulated gastric fluid.

**Figure 9:**
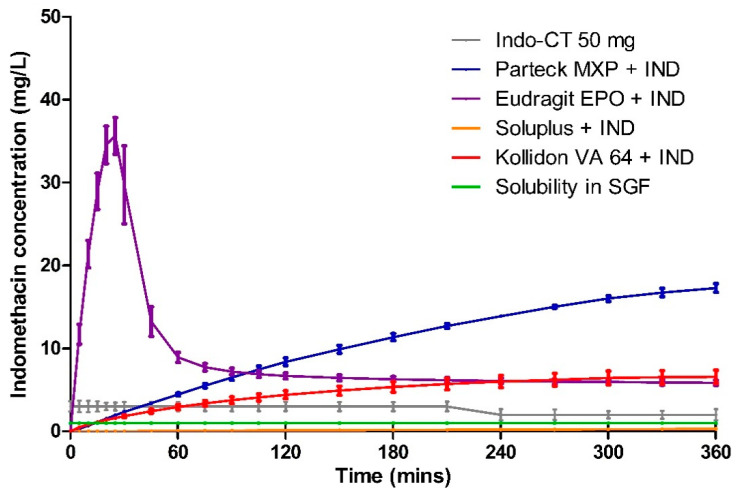
Dissolution testing for 8 mm VCM discs in simulated gastric fluid.

Dissolution data of milled VCM samples and milled HME samples are presented in Figure 7 and Figure 8, respectively. Figure 9 represents the dissolution data of the entire 8 mm VCM discs. Simulated gastric fluid (pH 1.2) was chosen to evaluate the supersaturation of indomethacin via the amorphous matrix. The 8 mm discs were directly used for the dissolution while the 20 mm samples were milled. The factors that played a vital role in the release behavior were the nature of the excipients and the surface area of the samples during the dissolution study. In the case of 8 mm discs, as they were placed intact in the dissolution medium, it had less surface area and hence very small surface was exposed to the dissolution medium to facilitate the drug release. While the 20 mm VCM and HME samples were milled, a large surface area was available for drug dissolution.

The similarity between the milled VCM and HME samples is shown using the *f2* similarity value. As per the FDA guidelines, the release profiles are considered similar when the *f2* value is greater than 50. If more than 85% of the drug is released, only a single value above that is considered [45]. However, in the case of our results, the steady-state concentration for all the formulations was found to be less than 85%. Hence, for the *f2* value calculations, we considered all the timepoints from the study. 

A very rapid onset can be observed for Eudragit^®^ EPO in the milled VCM and HME samples with a release of 45.04 ± 0.19 mg/L in 10 min and 46.00 ± 0.88 mg/L in 15 min, respectively. After an initial supersaturation for Eudragit^®^ EPO, precipitation occurs resulting in reduced concentrations. After 60 min, Eudragit^®^ EPO was able to maintain the drug concentration of at least 4.68 ± 0.20 mg/L for milled VCM samples and 6.53 ± 0.05 mg/L for milled HME samples until the end of the study. The observed release is due to the pH-sensitive solubility of Eudragit^®^ EPO in gastric juices up to a pH of 5.0 [46]. From the similarity factor (*f2* value) the drug release profile for milled VCM and HME samples is similar with the *f2* value of 50.79 (Table 6). 

**Figure 10:**
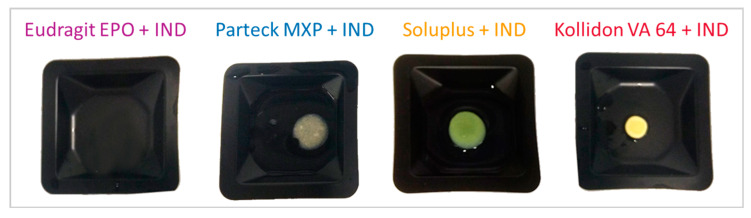
VCM discs after 6 h of dissolution on weighing boats.

For the Eudragit^®^ EPO 8 mm VCM discs, the highest concentration of 35.63 ± 2.21 mg/L was reached in 25 min. Similar to the milled samples, after an initial supersaturation, a stable plateau was observed at 105 min with a drug concentration of 6.88 ± 0.38 mg/L. The 8 mm VCM disc was completely dissolved by 360 min (Figure 10) as Eudragit^®^ EPO is highly soluble in pH 1.2, which correlates with an initial burst release.

Parteck^®^ MXP showed a peak drug concentration of 20.17 ± 0.70 mg/L at 120 min for milled VCM samples and then maintained a minimum drug concentration of more than 20.10 ± 0.98 mg/L until the end of 360 min. In the case of milled HME samples, the peak drug concentration of 23.02 ± 0.22 mg/L was observed at 60 min, and a drug concentration of more than 17.99 ± 0.29 mg/L was maintained. In contrast to Eudragit^®^ EPO, it can maintain the supersaturation for a longer timeframe. Due to its surface-active properties, polyvinyl alcohol of Parteck^®^ MXP can effectively stabilize the supersaturated state for a prolonged timeframe [47]. 

For the Parteck^®^ MXP 8 mm VCM discs, a sustained and incomplete release is observed with 17.28 ± 0.53 mg/L in 360 min owing to the diffusion behavior from the intact matrix of the disc. In the case of Parteck^®^ MXP milled HME samples and milled VCM samples had an *f2* value of 70.33, showing that the drug release profiles are similar.

In all the Kollidon^®^ VA 64 formulations, a supersaturation is observed but with limited drug release during the entire release period. With the highest drug release of 9.29 ± 0.47 mg/L for milled VCM samples, 7.40 ± 1.92 mg/L for milled HME samples, and 6.58 ± 0.79 mg/L for 8 mm VCM discs at 360 min. The observed results can be explained as Kollidon^®^ VA 64 at a pH of 1.2 and may preferentially dissolve from the matrix’s exterior by forming a drug-rich amorphous hydrophobic shell that inhibits the drug release [34]. As seen from Figure 10, the 8 mm VCM disc had retained its shape and did not show any disintegration for the drug release to occur. For the milled VCM and HME samples, an *f2* value of 84.59 was calculated, thus proving a similar release pattern.

Unlike the other excipients, extremely limited release from the Soluplus^®^ was observed. In the milled VCM samples, a release of 12.25 ± 0.69 mg/L, and for milled HME samples, a release of 3.44 ± 0.5 mg/L, was observed at 360 min. The *f2* value of 61.19 was obtained, proving a similar release behavior. For the 8 mm VCM discs, the negligible release was observed with a maximum release of 0.28 ± 0.05 mg/L. From Figure 10, we can see that Soluplus^®^ shows water absorption and swelling while retaining the disc-like shape. The limited release from Soluplus^®^ can be attributed to the possible formation of hydrogen bonds between the carboxylic groups on indomethacin and oxygen atoms in the Soluplus^®^. Due to the formation of hydrogen bonding at pH 1.2, there is a reduction in the solubility of the polymer leading to decreased release [48]. Furthermore, in the case of an 8 mm VCM disc, the surface area available for drug release is limited, while in the case of milled samples, the surface area is increased multi-folds. This increase in the surface area can explain the observed higher drug release from milled samples despite low solubility.

Compared to other polymers, PVA shows at least a four times higher drug release than Soluplus^®^, Kollidon^®^ VA 64, and the marketed formulation Indo-CT 50 mg. Furthermore, compared to the solubility of the pure indomethacin, Parteck^®^ MXP enhanced the release by almost 20 times. 

The area under the curve (AUC) was calculated using the trapezoidal rule for the release profiles of milled HME, milled VCM, 8mm VCM disc, and the marketed product Indo-CT 50 mg (Table 7). Considering the obtained AUC results of milled VCM, milled HME, and 8mm discs, we can establish an overall ranking of the polymers for ASD. From all the polymers, Parteck^®^ MXP showed the highest maintained supersaturation levels for indomethacin in the case of milled as well as 8mm disc samples. It was followed by Eudragit^®^ EPO, Kollidon^®^ VA 64, and then Soluplus^®^. The high variability of AUCs observed for Soluplus^®^ can be attributed to the factors mentioned above like available surface area and hydrogen bonding. Collectively, if we compare the results obtained via the VCM tool to the results of the hot-melt extruded formulations in Figure 7 and Figure 8, a remarkably similar pattern can be observed. This highlights the high predictability of the MeltPrep^®^ VCM technology. The performance of different polymers can be assessed at a low sample size and provide high reliability of prediction.

## 4. Conclusions

In this study, the MeltPrep^®^ VCM technology was assessed as a potential screening tool for melt-based formulations at a small scale, where we also demonstrated the loss-less processing. 

Indomethacin as a model drug was utilized for the preparation of solid dispersions using HME and VCM technology. The primary objective of the study was to compare the formation of ASDs to enhance the solubility of indomethacin and to assess the similarity between the two technologies. It was determined through the comparison of thermal analysis, PXRD, and dissolution profiles. From the DSC thermograms and PXRD diffractograms, we confirmed that an amorphous solid dispersion was formed using both the technologies. This was further proved by similar dissolution profiles indicating the comparability of the VCM samples to that of the HME. Hence, proving our objective of utilizing VCM as an explorative tool for HME-based formulations. With a fast preparation time, small sample amount requirements, and with choice of different sample sizes and shapes, VCM will be a feasible predictive tool for extruded formulations, especially on a small scale.

We could demonstrate the application of VCM technology for a set of widely used polymers in HME, especially showing a clear advantage for hydrophilic polymers, which would be potentially excluded by a standard film casting screening. Further processing of VCM samples such as milling can mimic particle properties as they would be obtained from milled extrudates enabling down-streaming to a conventional tablet design.

When comparing the efficiency, Parteck^®^ MXP showed better results among all selected polymers. It was able to show a sustained drug release as well as maintained a steady-state concentration at a much higher level compared to other polymers. Even though Eudragit^®^ EPO showed an initial burst release, the drug concentration quickly dropped down and was sustained at a very low value compared to initial supersaturation concentration. While Soluplus^®^ and Kollidon^®^ VA 64 showed a drug release of less than 20% in 360 min.

One advantage of the early screening technology is to identify potential interactions between drug substances and test polymers at early development stages like the observed low drug release in the case of Soluplus^®^. VCM utilizes small quantities of material compared to HME, thus preventing the wastage of material in early development screening.

A visual analysis of the samples during the dissolution process can provide important insight into the release mechanism. Understanding molecular interactions within the matrix can support the identification of the best possible carrier at the early stages.

## Figures and Tables

**Table 1:** Physicochemical properties of model drug.

API	M_w_ (g/mol)	logP	T_m_ (°C)	T_g_ (°C)	pKa	SGF Solubility [37]
Indomethacin	357.8	4.27	155 ± 0.1	49 ± 0.1	4.5	0.004 g/1000 g

**Table 2:** Physicochemical properties of polymers used in amorphous solid dispersions (ASD).

Polymer	Classification	Average Molecular Weight (g/mol)	Glass Transition (°C)	Degradation Temperature (°C)	Water Solubility (pH-7)
Parteck^®^MXP [38]	Non-ionic	32,000	54	250	Soluble
Soluplus^®^ [39]	Non-ionic	118,000	65–70	250	Soluble
Kollidon^®^VA-64 [39]	Non-ionic	45,000	100	230	Soluble
Eudragit^®^ EPO [40]	Ionic	47,000	48	200	Insoluble

**Table 3:** Processing parameter of VCM samples.

Polymers	20 mm VCM Disc Insert		8 mm VCM Disc Insert	
	Heating Temperature (°C)	Heating Time (min)	Heating Temperature (°C)	Heating Time (min)
Parteck^®^ MXP	230	5	230	5
Soluplus^®^	170	5	170	4
Kollidon^®^ VA-64	160	5	160	4
Eudragit^®^ EPO	190	5	190	4

**Table 4:** Hot-melt extrusion (HME) process parameters.

Polymer	Pressure (bar)	Melt Temperature (°C)	Barrel Temperature for All The Zones (°C)	Torque % of Max.	Torque (Nm)
Parteck^®^ MXP	8–10	181	190	20	1–2
Soluplus^®^	1	151	160	24	1–4
Kollidon^®^ VA 64	0–1	152	160	40	2–4
Eudragit^®^ EPO	0	152	160	43	2–6

**Table 5:** Lossless preparation of MeltPrep^®^ VCM samples (*n* = 3).

Excipients	Before VCM Process Weight (mg)		After VCM Process Weight (mg)	
	8 mm	20 mm	8 mm	20 mm
Parteck^®^ MXP	167.51 ± 0.29	498.35 ± 0.84	166.46 ± 0.39	495.75 ± 1.77
Soluplus^®^	168.67 ± 1.21	502.43 ± 1.27	166.90 ± 0.56	499.20 ± 0.31
Kollidon^®^ VA 64	167.23 ± 2.05	500.85 ± 1.57	165.47 ± 2.56	498.98 ± 1.68
Eudragit^®^ EPO	167.24 ± 1.78	500.12 ± 0.45	165.37 ± 1.08	497.39 ± 1.32

**Table 6:** Similarity factor (*f2*) for the VCM 20 mm samples and HME samples.

Polymer	*f2* Value
Parteck^®^ MXP	70.33
Soluplus^®^	61.19
Kollidon^®^ VA 64	84.59
Eudragit^®^ EPO	51.78

**Table 7:** Area under the curve (AUC) ± RSD for release profiles of HME and VCM samples (*n* = 3).

Polymer	Milled HME (mg·L^−1^·min)	Milled VCM (mg·L^−1^·min)	8 mm VCM (mg·L^−1^·min)
Parteck^®^ MXP	7196.02 ± 1.09	6940.29 ± 3.96	3778.45 ± 1.25
Soluplus^®^	1053.25 ± 14.22	3421.46 ± 7.52	57.31 ± 22.96
Kollidon^®^ VA 64	1752.93 ± 25.91	2459.68 ± 6.43	1725.85 ± 14.17
Eudragit^®^ EPO	3855.90 ± 5.74	2557.37 ± 2.38	3140.94 ± 7.94

Marketed Product—Indo-CT 50 mg—933.33 ± 20.86 mg·L^−1^·min.
